# Comparative analysis of metabolites in different *Areca* inflorescence tissues based on spatial metabolomics

**DOI:** 10.3389/fpls.2025.1687705

**Published:** 2026-01-30

**Authors:** Xinyu Wen, Panjing Li, Linbi Zhang, Han Li, Fusun Yang

**Affiliations:** Country School of Tropical Agriculture and Forestry, Hainan University, Haikou, Hainan, China

**Keywords:** alkaloids, *Areca catechu*, floral organs, sex differentiation, spatial metabolomics

## Abstract

In this study, we focused on the spatial distribution of metabolites in areca palm (*Areca catechu* L.) floral organs. Using spatial metabolomics, the composition and accumulation patterns of active metabolites were delineated to provide a basis for the development and utilization of their bioactive compounds. The present study combined untargeted metabolomics and mass spectrometry imaging, enabling high-resolution spatial visualization of metabolites in floral tissue microregions, with an emphasis on alkaloids, flavonoids, coumarins, and cinnamic acid. Significant differences in metabolite contents were observed using statistical methods and spatial metabolic mass spectrometry, revealing how these metabolites clustered and varied across tissues. Areca alkaloids exhibited specific enrichment in the ovary locules and ovule tissues of female flowers, showing concentrations 3–5 times higher than those in other tissues. Flavonoids were primarily localized in the vascular bundle sheath cells of the ovary wall. Although coumarins and cassia bark acids were distributed across all four tissue types, they displayed a gradient distribution pattern in the epidermal layer of female flower ovules. Using spatial metabolomics, this research reveals the compartmentalized distribution of metabolites across *A. catechu* floral organs, shedding light on their tissue-specific functions and related metabolic pathways.

## 1 Introduction

Areca palm (*Areca catechu* L.), a tropical and traditional medicinal plant of economic value, is known for its areca nuts, which are rich in secondary metabolites such as alkaloids, flavonoids, coumarins, and cassia bark acids (Romadhon et al., 2024; Yu et al., 2023). *Areca catechu* inflorescences are a traditional food resource in Hainan Province, China, and they are also rich in polyphenols, alkaloids, flavonoids, and other active ingredients (Frost, 2023; Wang and Luo, 2024). Research has shown that these inflorescences have physiological activities, such as lowering blood glucose and blood lipid cholesterol, antioxidant and antibacterial properties, and pharmacological activities, such as cough suppression and the promotion of gastric health, giving them potential for development and utilization (Bai et al., 2020; Zhao et al., 2023).

Research on metabolites in *A. catechu* flowers has increased through the use of multi-level analytical technologies. Early spectrophotometric methods confirmed the richness of phenolic and flavonoid compounds in these flowers, with boiling water extracts exhibiting significantly higher total phenolic content than organic solvent extracts; the roasting process effectively preserves their active components (Lin and Li, 2010; Zhang et al., 2009). Chromatography-mass spectrometry enables precise quantification. High-performance liquid chromatography analysis with diode-array detection revealed that arecoline content peaked at the initial flowering stage and identified six characteristic polyphenols (Jantarat et al., 2013; Wang et al., 2011). Gas chromatography-mass spectrometry further detected 46 metabolites in male flowers, showing a higher relative abundance of nitrogen-containing compounds compared with female flowers. Multi-omics technologies have further unveiled related mechanisms (Xu et al., 2024). Untargeted metabolomics identified 331 metabolites in areca inflorescences, with the flavonoid content in root and leaf tissues being 3.2-fold higher than that in flowers, and they co-localized with 4746 differentially expressed genes (Lai et al., 2023). Spatial metabolomics achieved *in situ* visualization of the gradient distribution of 35 metabolites in areca rhizomes, confirming alkaloid enrichment in epidermal tissues and the specific accumulation of flavonoid glycosides in vascular bundles (Wang J. et al., 2024).

This study aimed to overcome the technical bottleneck of spatial metabolite localization by integrating untargeted metabolomics and mass spectrometry imaging, thereby constructing a spatial expression atlas of metabolites in *A. catechu* floral organs (Ries et al., 2023; Wang J. et al., 2024). This study employed high-resolution imaging to achieve microregional visualization at the tissue level, focusing on four classes of functional metabolites: alkaloids, flavonoids, coumarins, and cassia bark acids (Li et al., 2019). By systematically comparing the metabolic differences among female and male flowers, female peduncles, and inflorescence structures, we aimed to identify tissue-specific enrichment patterns in various metabolite classes using spatial metabolomics (Li et al., 2025).

This study systematically revealed the compartmentalized distribution patterns of metabolites in *A. catechu* floral tissues, including the accumulation of areca alkaloids in the ovary locules, the exact position of flavonoids in vascular bundles, and the gradual distribution of coumarins across the ovule epidermis. As the first spatial metabolomics study of *A. catechu* floral organs, this work offers initial insights into the tissue-specific localization of secondary metabolites, paving the way for future research on their biological functions and exploitation.

## 2 Materials and methods

### 2.1 Plant materials

The experimental materials comprised *A. catechu* flowers obtained from the experimental base of Hainan University’s Danzhou campus (located at 19°30’27”N, 109°29’3”E, China). *Areca catechu* flower samples were collected from the second leaves of a 10-year-old tree.

### 2.2 Pre-treatment of *A*. *catechu* samples for MALDI analysis

**Figure 1:**
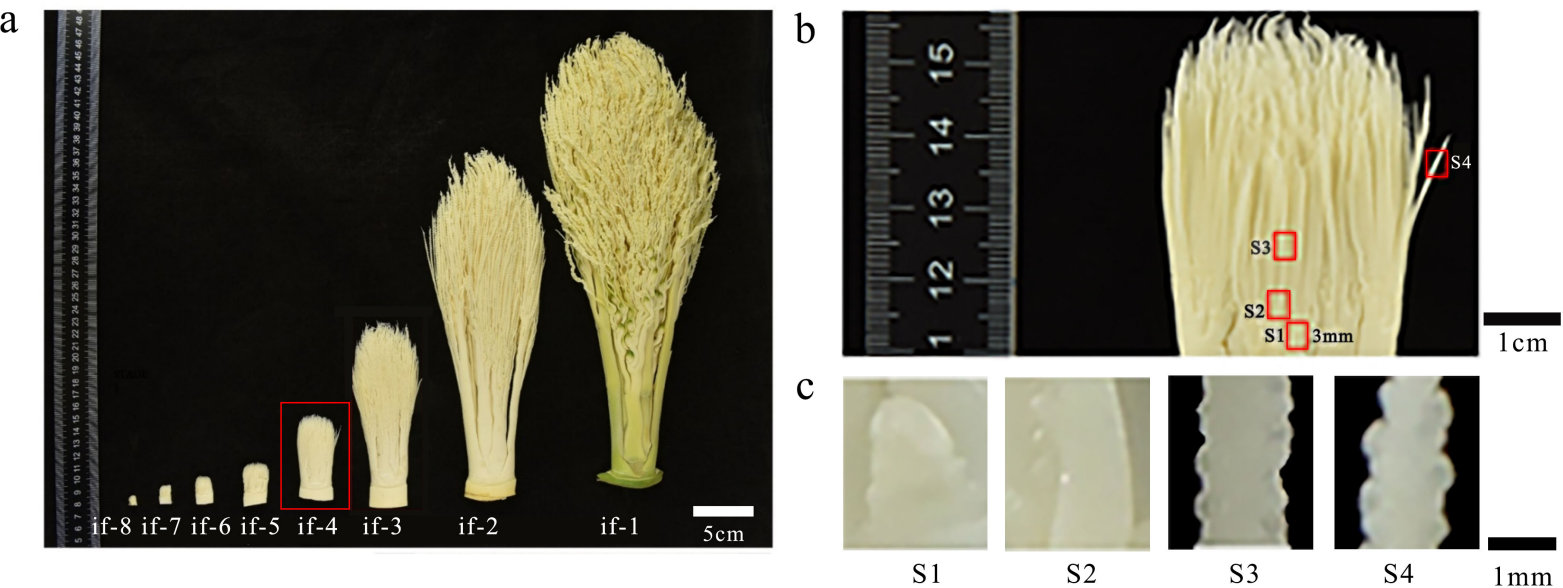
*Areca catechu* L. flower sample preparation methods. **(a)** Schematic diagram of the source site of if-4. **(b)** Schematic diagram of the sample distribution. S1, S2, S3, and S4 are female flowers, male flowers, female peduncle, and inflorescence, respectively. **(c)** Schematic diagram of the local sample. The female flower is about 1.2–1.5 mm in size, and the male flower just protrudes.

Based on continuous observation and investigation of the inflorescence development in adult areca palms, preliminary findings indicate a marked developmental transition from the inflorescence-4 to the inflorescence-3 stage, characterized by a rapid increase in the size and weight of flower buds at the inflorescence-3 phase. It is thus hypothesized that inflorescence-4 likely represents a critical period for sex differentiation, whereas inflorescence-3 primarily corresponds to the stage of rapid floral organ growth (**Supplementary Table S1**).As shown in **Figures 1a–c**, the fourth period of inflorescence development (if-4) was selected. The inflorescence extends from the position where female flowers appear next to the male flowers (approximately 3.5–4.5 mm).Based on the significant variation in the number of male and female flowers among different inflorescences, a comparative analysis was conducted between inflorescences with developed flowers and undifferentiated inflorescences. This approach aims to uncover, at the metabolomic level, the potential mechanisms underlying the differential development of male and female flowers, as well as the failure of floral primordia to form normal unisexual flowers in certain inflorescences. It was cut into four segments from top to bottom: female flower, male flower, female peduncle, and inflorescence. Each segment was then cut into small pieces with a diameter of 2 mm and a height of 3 mm (labeled as S1–S4 in the following figure). Cryosectioning of the tissue samples was performed using a Leica CM1950 cryostat at a thickness setting of 10–20 μm. The resulting sections were transferred onto specialized conductive glass slides compatible with MALDI-2 using a pre-cooled brush. A matrix solution suitable for MALDI-2 applications was prepared and uniformly applied onto the tissue-bearing slides using a TM-Sprayer matrix coating system (Bagri et al., 2024).

The matrix spraying parameters mainly included a matrix of 9-AA, a solvent composed of 70% ethanol + 30% water, a nozzle temperature set at 90°C, a nozzle speed of 1200 mm/min, a flow rate of 0.12 ml/min, a nitrogen pressure of 10 psi, and a track spacing of 2 mm (Brunetti et al., 2020).

### 2.3 Mass spectrometry imaging analysis

The coated slides were then mounted onto the target plate of a Timstof flex MALDI 2 instrument. Using Bruker Data Imaging software, the regions of interest on the tissue sections were selected, and the imaging resolution was defined. According to the dimensions of each section, the area was divided into a two-dimensional array of measurement points for imaging. Under appropriate laser energy, the tissue sections were scanned, and molecules released via ionization from specific sites were detected by mass spectrometry, yielding raw mass spectral data files.

The mass spectrometry imaging parameters mainly included a metabolite mass-to-charge ratio detection range of 50–1000 m/z, an ion detection mode of negative ion, an imaging resolution of 50 μm, a laser frequency of 10000 Hz, a laser energy of 70%, and a number of acquisitions per pixel of 200.

### 2.4 Data analysis

Initially, a univariate analysis method was used to perform differential tests on each metabolite individually. The results of the univariate analysis were further supported and visualized using Principal Component Analysis (PCA). The PCA score plot provided a multivariate perspective on sample grouping, revealing whether the statistically significant metabolites identified through univariate tests contributed to the observable separation between sample classes. The original p-values were calculated using t-tests, and the Benjamini-Hochberg false discovery rate (FDR) correction was applied to control for multiple comparisons. The fold change (FC) of each metabolite was log2-transformed to obtain log_2_FC values. A threshold of |log_2_FC| > 1 was set to screen for metabolites with significant abundance changes. The relationship between log_2_FC and the corresponding FDR-corrected p-values was visualized using a volcano plot (Li et al., 2025), which facilitated the identification of key differentially expressed metabolites. The following screening criteria were established: metabolites with log_2_FC ≥ 0.5 were up-accumulated, and log_2_FC ≤ −0.5 were downregulated.

The tissue sections were subjected to mass spectrometry imaging analysis, and the acquired raw data were processed using SCiLS™ Lab 2021 software, which included procedures for baseline subtraction, peak alignment, spectral smoothing, and normalization. Peak picking and metabolite identification were subsequently performed on the processed mass spectrometry data with the Bruker Metaboscape™ Workstation. Metabolite annotation was conducted by matching experimental results against both an in-house database and the Bruker Library MS-Metabobase 3.0, applying a mass accuracy threshold of <10 ppm for theoretical m/z values (Wu et al., 2019).

## 3 Results

### 3.1 General overview of spatial differences in metabolite profiles

**Figure 2:**
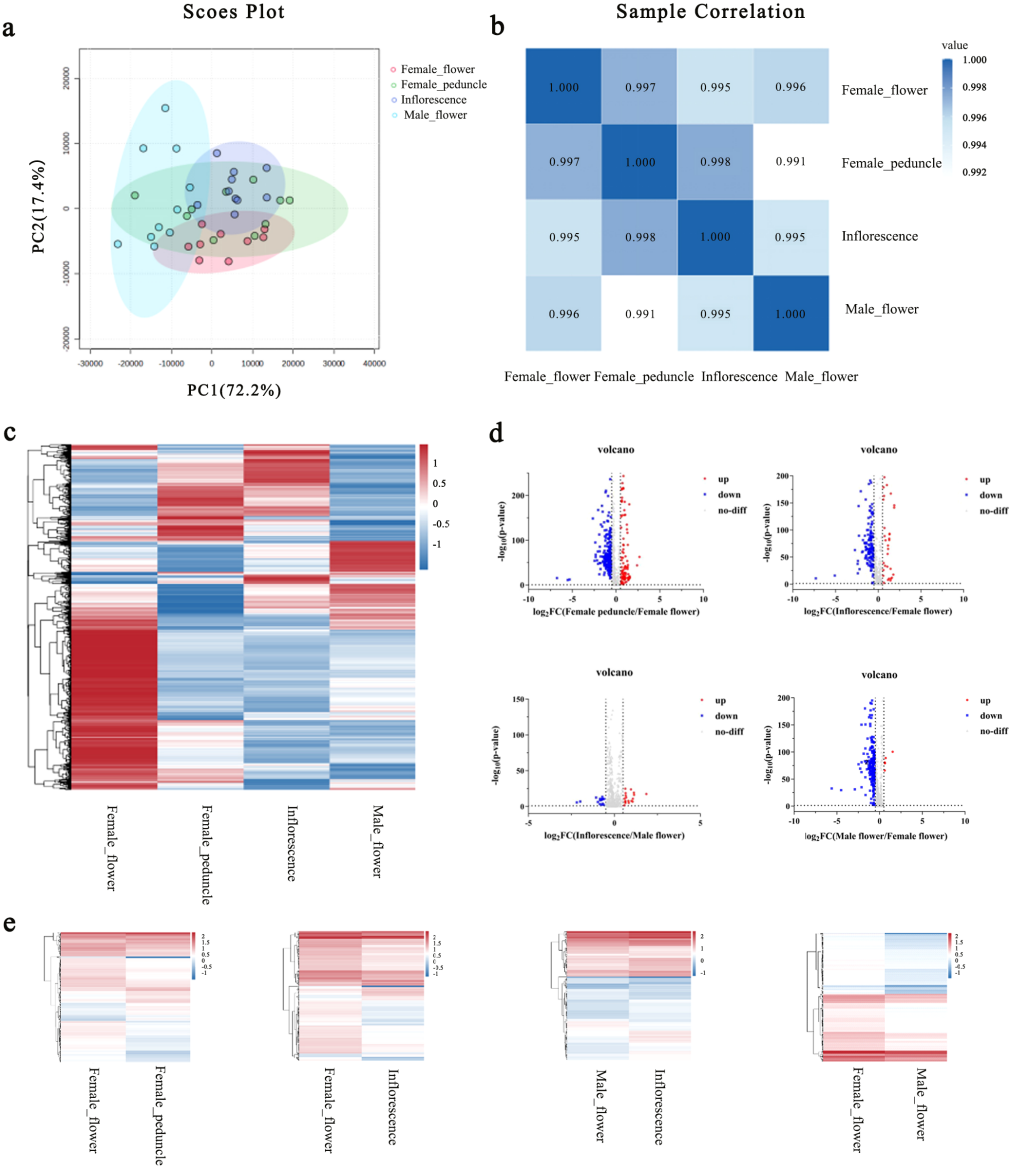
Graphical representation of the overall differential metabolites in the samples. **(a)** PCA scores of mass spectrometry data from four groups and quality control samples. **(b)** Correlation coefficients for the four sample groups. **(c)** Overall cluster heat map of samples. **(d)** Sample grouping volcano map analysis. Red represents upregulated metabolites, blue represents downregulated metabolites, and gray represents metabolites with no significant changes. **(e)** Clustering heat map for the four sample groups.

Principal Component Analysis (PCA) was employed to visualize the overall metabolic distribution and to assess the intrinsic variability between samples. In the PCA score plot (**Figure 2a**), each point represents an individual sample. The first two principal components, PC1 and PC2, effectively captured the structure of the raw LC-MS data, accounting for 72.2% and 17.4% of the total variance, respectively. Cumulatively, they explained 89.6% of the variance, indicating that these two dimensions sufficiently represent the major metabolic characteristics of the *A. catechu* flower samples. In **Figure 2b**, the legend represents the correlation coefficient between different samples. The correlation coefficient between samples was close to 1, indicating that the data were well controlled, the instrument was stable during testing, and the data detected were of high quality. Based on cluster heatmap analysis (**Figure 2c**), the four sample groups differed, while the components of the parallel samples within the groups were similar, demonstrating the reliability of the sample data. Significant differential metabolites were selected based on the results of relative variable analysis, with FDR-corrected p-values < 0.05. The log_2_FC of up-accumulated metabolites was greater than or equal to 0.5, while that of downregulated metabolites was less than or equal to −0.5. Among the examined *A. catechu* inflorescences, the female flowers, male inflorescences, and male flowers were grouped. As shown in **Figure 2d**, there were 169 differential metabolites between the female flower and female peduncle, 182 between the female flower and inflorescence, 86 between the male flower and inflorescence, and 121 between the female flower and male flower. The log_2_FC among differential metabolites was normalized. As shown in **Figure 2e**, the four groups were well differentiated from the metabolite types. **Tables 1**–**4** show that the material species and relative content of the differential metabolites changed. Comparison groups female flower vs female peduncle and female flower vs inflorescence revealed differential metabolites (**Tables 1**, **2**), including cassia bark acids, coumarin, and lipids, and significantly differential metabolites, including alkaloids, flavonoids, benzoether, nucleosides, nucleotides and their analogues, organic heterocyclic compounds, and organic oxygen compounds. Between female and male flowers (**Table 3**), the significantly downregulated metabolites were alkaloids and their derivatives, flavonoids, cassia bark acids and their derivatives, coumarins and their derivatives, organic acids and their derivatives, lipids, benzoic acids, nucleosides, nucleotides and their analogs, organic heterocyclic compounds, and organic oxygen compounds. Of the differential metabolites between male flowers and inflorescence (**Table 4**), flavonoids, organic acids, and lipids were significantly up-accumulated, and alkaloids and their derivatives, cassia bark acids and their derivatives, and benzoic acids were significantly downregulated.

**Table 1:** Differences in metabolite types and changes between female flowers and female peduncles.

Order number	Super class classification	Total	Up	Down
1	Alkaloids and derivatives	1	0	1
2	Flavonoids	6	1	5
3	Cinnamic acids and derivatives	3	3	0
4	Coumarins and derivatives	4	4	0
5	Organic acids and derivatives	18	7	11
6	Lipids	31	31	0
7	Benzenoids	32	1	31
8	Nucleosides, nucleotides, and analogues	17	1	16
9	Organoheterocyclic compounds	28	3	25
10	Organic oxygen compounds	14	0	14

**Table 2:** Differences in metabolite types and changes between female flowers and inflorescences.

Order number	Super class classification	Total	Up	Down
1	Alkaloids and derivatives	1	0	1
2	Flavonoids	4	0	4
3	Cinnamic acids and derivatives	3	3	0
4	Coumarins and derivatives	6	6	0
5	Organic acids and derivatives	23	4	19
6	Lipids	26	23	3
7	Benzenoids	41	1	40
8	Nucleosides, nucleotides, and analogues	20	1	19
9	Organoheterocyclic compounds	31	1	30
10	Organic oxygen compounds	15	0	15

**Table 3:** Metabolite types and changes between female and male flowers.

Order number	Super class classification	Total	Up	Down
1	Alkaloids and derivatives	1	0	1
2	Flavonoids	5	0	5
3	Cinnamic acids and derivatives	1	0	1
4	Coumarins and derivatives	3	0	3
5	Organic acids and derivatives	14	0	14
6	Lipids	3	0	3
7	Benzenoids	33	1	32
8	Nucleosides, nucleotides, and analogues	13	0	13
9	Organoheterocyclic compounds	26	0	26
10	Organic oxygen compounds	9	0	9

**Table 4:** Changes in metabolites between male flowers and inflorescences.

Order number	Super class classification	Total	Up	Down
1	Alkaloids and derivatives	1	0	1
2	Flavonoids	5	4	1
3	Cinnamic acids and derivatives	1	0	1
4	Coumarins and derivatives	2	1	1
5	Organic acids and derivatives	6	5	1
6	Lipids	29	29	0
7	Benzenoids	9	1	8
8	Nucleosides, nucleotides, and analogues	5	2	3
9	Organoheterocyclic compounds	11	3	8
10	Organic oxygen compounds	7	3	4

### 3.2 Metabolite changes in *A. catechu* flowers

There were numerous active ingredients in *A. catechu*. The primary focus of this study was on the relative content changes in flavonoids, alkaloids, cassia bark acids, and coumarins. There were eight differential metabolites in the four comparison groups: 1 alkaloid (brasofensine), 3 flavonoids (oolongtheanin, 3-hydroxy-8,9-methylenedioxypterocarpane, and neobignonoside), 3 coumarins (daphnoretin, 4-methylumbelliferyl glucuronide, and 4-methylumbelliferone sulfate), and 1 cinnamic acid (caftaric acid). Alkaloids were active substances in *A. catechu* inflorescences. The changes among alkaloids in the four tissues are shown in **Table 5**. The alkaloid brasofensine was detected and had the highest content in female flowers and the lowest content in inflorescences.

**Table 5:** Differences in alkaloid metabolites between female flowers, male flowers, female peduncles, and inflorescences.

Order number	mz	Metabolites	FF	FP	I	MF
1	325.0896 m/z ± 0.01 Da	Brasofensine	148.535614	49.8832588	40.7220192	71.6337357

In the study of flavonoid metabolites, differential compounds with FDR-corrected p-values (< 0.05) were identified across floral developmental stages (stage if-4), including between female flowers and peduncles, female flowers and inflorescence, female and male flowers, and male flowers and inflorescences, indicating their importance in the biological processes of the plant. **Table 6** shows that the contents of oolongtheanin, 3-hydroxy-8,9-methylenedioxypterocarpane, neobignonoside, daidzein 4’-sulfate, sissotrin, and other substances were highest in female flowers. Isorhamnetin 3-sulfate, piraeoside, 3,5,6-trihydroxy-3′, and 4′,7-trimethoxyflavone 3-glucuronide were highly expressed in female peduncles.

**Table 6:** Differences in flavonoid metabolites between female flowers, male flowers, female peduncles, and inflorescences,.

Order number	mz	Metabolites	FF	FP	I	MF
1	713.1186 m/z ± 0.01 Da	Oolongtheanin	1.75981557	0.038385414	0.052019503	0.236662611
2	283.0662 m/z ± 0.01 Da	3-Hydroxy-8,9-methylenedioxypterocarpane	8.46473026	4.83693123	4.60838366	5.64070702
3	567.1107 m/z ± 0.01 Da	Neobignonoside	3.85349751	0.706976593	0.579537213	0.701505065
4	333.0072 m/z ± 0.01 Da	Daidzein 4'-sulfate		4.07090998	3.84168053	
			5.53428698			3.45225692
	
5	392.9887 m/z ± 0.01 Da	Isorhamnetin 3-sulfate	16.8209915	24.1386433		
					24.1688633	14.3131599
	
6	463.0864 m/z ± 0.01 Da	Spiraeoside	32.1552849	43.8359261	42.7606544	27.4557495
7	535.1074 m/z ± 0.01 Da	3,5,6-Trihydroxy-3',4',7-trimethoxyflavone 3-glucuronide	42.7059708	62.9414635	61.0627747	35.5742149
8	587.1263 m/z ± 0.01 Da	Kolaflavanone	2.81178665	5.05301809	3.18408632	3.94649076
9	445.1116 m/z ± 0.01 Da	Sissotrin	6.03495312	3.62108207	4.27903461	4.24627304

For coumarin acids, differential metabolites between female flowers and female peduncles, female flowers and inflorescence, female flowers and male flowers, and male flowers and inflorescences with FDR-corrected p-values < 0.05 were selected. As shown in **Table 7**, daphnoretin, 4-methylumbelliferyl glucuronide, 4-methylumbelliferone sulfate, 7-hydroxycoumarin sulfate, and isopimpinellin showed the highest accumulation in female flowers and the lowest accumulation in inflorescences.

**Table 7:** Differences in coumarin metabolites between female flowers, male flowers, female peduncles, and inflorescences.

Order number	mz	Metabolites	FF	FP	I	MF
1	351.0552 m/z ± 0.01 Da	Daphnoretin	6.94858742	3.65946507	3.45241737	4.11293888
2	351.0753 m/z ± 0.01 Da	4-Methylumbelliferyl glucuronide	16.6432819	10.255722	9.93672562	10.810812
3	236.9861 m/z ± 0.01 Da	4-Methylumbelliferone sulfate	2.66145468	1.36804533	1.09552431	1.37752688
4	240.9821 m/z ± 0.01 Da	7-hydroxycoumarin sulfate	4.908689	4.25583363	3.1586225	3.40102673
5	281.0167 m/z ± 0.01 Da	Isopimpinellin	5.785602	3.63995504	3.06974125	4.2154417

For cassia bark acids, differential metabolites with FDR-corrected p-values < 0.05 between female flowers and female peduncles, female flowers and inflorescence, female flowers and male flowers, and male flowers and inflorescences were selected. As shown in **Table 8**, osmanthuside A, rosmarinic acid, 2-O-p-coumaroyltartronic acid, and caftaric acid showed the highest accumulation in female flowers.

**Table 8:** Differences in cassia bark metabolites between female flowers, male flowers, female peduncles, and inflorescences.

Order number	mz	Metabolites	FF	FP	I	MF
1	427.1418 m/z ± 0.01 Da	Osmanthuside A	18.7429371	11.300415	11.4890766	13.0333548
2	359.0772 m/z ± 0.01 Da	Rosmarinic acid	6.558494	5.1186738	3.60147333	4.63909149
3	265.0351 m/z ± 0.01 Da	2-O-p-Coumaroyltartronic acid	5.72134066	3.35173917	3.68542314	4.07065201
4	311.0395 m/z ± 0.01 Da	Caftaric acid	6.38586569	3.33064604	2.58184385	3.91171217

### 3.3 Spatial metabolomic atlas in *A*. *catechu* floral tissues

**Figure 3:**
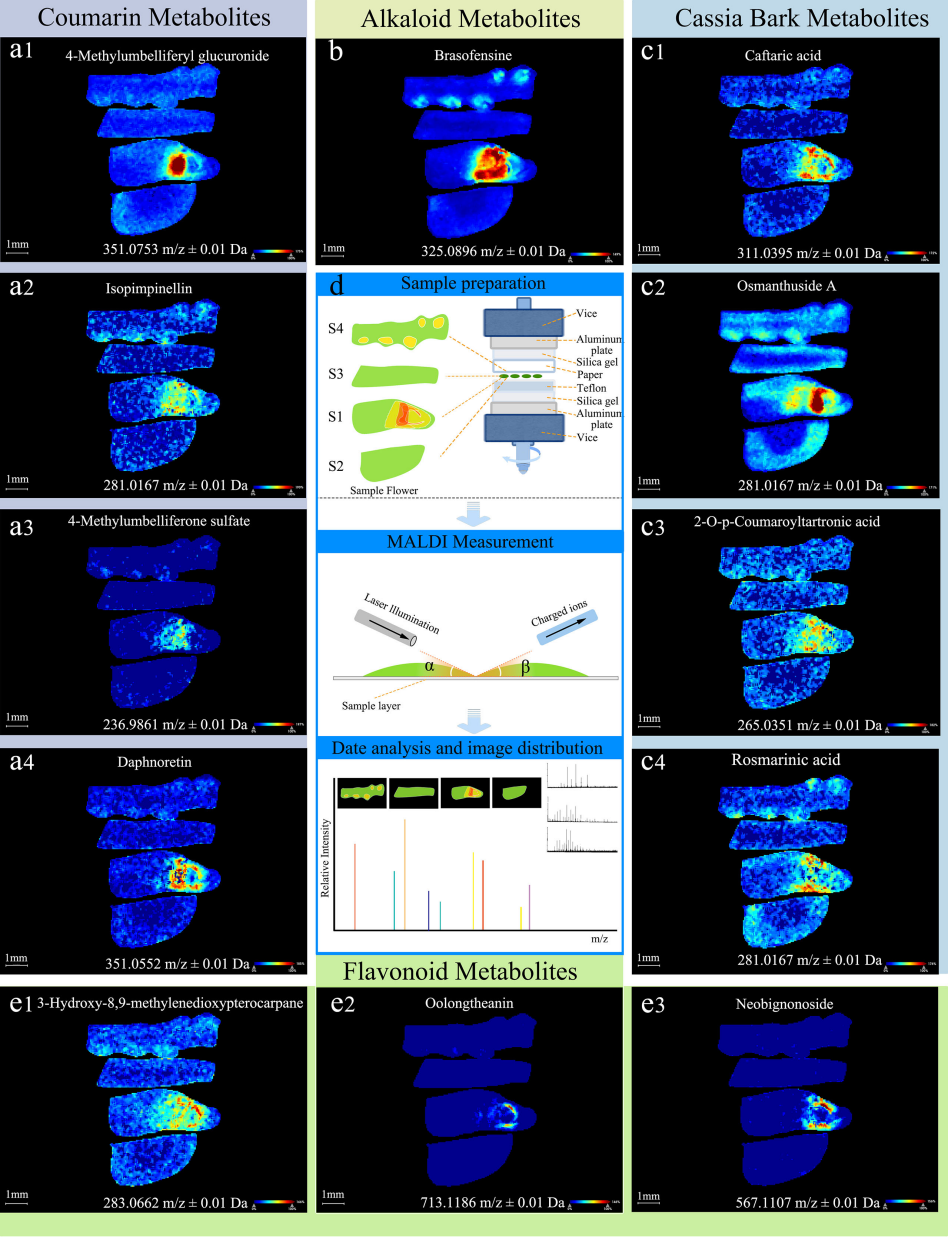
Within-tissue spatial distributions of small molecules on both sides of *Areca catechu* flowers. **(a1–4)** Differences in alkaloid metabolites between female and male flowers, female peduncles, and inflorescences. **(b)** Differences in flavonoid metabolites between female and male flowers, female peduncles, and inflorescences. **(c1–4)** Differences in coumarin metabolites between female and male flowers, female peduncles, and inflorescences. **(d)** MALDI imaging experiment workflow. S1, S2, S3, and S4 are female flowers, male flowers, female flower stalks, and inflorescence, respectively. **(e1–3)** Differences in cassia bark metabolites between female and male flowers, female peduncles, and inflorescences.

Based on the differential metabolites identified through the aforementioned untargeted metabolomics approach, a total of 12 metabolites were matched to their corresponding spatial mass spectrometry images according to their compound names and accurate mass-to-charge ratios (m/z). As shown in **Figure 3**, these metabolites are primarily classified into four categories: the alkaloid metabolite brasofensine; the flavonoid metabolites 3-Hydroxy-8,9-methylenedioxypterocarpane, Oolongtheanin, and Neobignonoside; the coumarin metabolites 4-Methylumbelliferyl glucuronide, Isopimpinellin, 4-Methylumbelliferone sulfate, and Daphnoretin; and the cassia bark metabolites Caftaric acid, Osmanthuside A, 2-O-p-Coumaroyltartronic acid, and Rosmarinic acid.

## 4 Discussion

### 4.1 Compartmentalized metabolites in *A*. *catechu* floral tissues

Metabolomics studies revealed the compartmentalized distribution of metabolites within A. catechu floral tissues, showing a rich variety of bioactive substances, such as alkaloids, polyphenols, pectins and vitamin C, as well as amino acids and various inorganic elements. This study comprehensively analyzed the spatial distribution patterns of four key classes of functional metabolites—alkaloids, flavonoids, coumarins, and cassia bark acids—in *A*. *catechu* floral tissues, namely, female and male flowers, female peduncles, and inflorescences (Wu et al., 2021, 2019). Integrated analyses revealed an uneven distribution of metabolites across the floral tissues, with distinct tissue-specific enrichment patterns (Lai et al., 2023). These patterns reflect potential correlations between metabolite localization and tissue function and underscore the compartmentalized nature of the underlying biosynthetic and regulatory networks (Wu et al., 2022).

### 4.2 Enrichment of alkaloids in female flowers suggests a reproductive defense function

Alkaloids (brasofensine) showed different distributions in the four parts of the *A. catechu* inflorescence and were distributed in female and male flowers (**Figure 3b**) (Alseekh et al., 2021). In female flowers, they predominantly localize to the ovary and ovule tissues, a pattern associated with reproductive structures and consistent with findings in species like tobacco (*Nicotiana tabacum*) (Ding et al., 2023; Lai et al., 2022). The distribution of alkaloids in female flowers is related to reproductive-related structures (e.g., ovary), while alkaloids are concentrated in pollen grains and petal fiber bundles in male flowers (Li et al., 2025). This difference may be related to its biological function, as alkaloids in female flowers may participate in ovule protection or seed development regulation. High alkaloid concentrations may inhibit pathogens or pests in reproductive tissues, act as signaling molecules, or participate in the sex differentiation process mediated by jasmonic acid (JA) (Li et al., 2024).

### 4.3 Flavonoids localize to vascular bundle tissues

The specific localization of flavonoids in vascular bundle indicates their involvement in metabolite transport or signaling pathways, consistent with their known roles in regulating auxin flow and vascular development in plants. Using spatial metabolomics techniques, the analysis of *A*. *catechu* flowers revealed that flavonoids such as oolongtheanin extract and neobigenin (neobignonoside) exhibit a significant spatially specific distribution in female flowers. This study showed that these two compounds are almost entirely confined to female flowers (**Figure 3e**(**1–3**)), showing high concentrations in the vascular bundles of the ovary wall in the pistil, and they are hardly detectable in male flowers or other vegetative tissues (Qi et al., 2025; Wang C. et al., 2024). This distribution pattern is closely related to the antioxidant protection needs and pathogen defense mechanisms of female reproductive organs and aligns with the preferential distribution patterns of flavonoids in reproductive tissues of palms, indicating the evolutionary conservation of their functions (Ansari et al., 2021).

### 4.4 Gradient distribution of coumarins and cassia bark acids suggests their association with developmental stage-specific processes

Coumarins accumulate significantly in both female and male flowers of *A*. *catechu*, exhibiting a gradual difference in concentration. **Figure 3a** (**1–4**) demonstrates that 4-methylumbelliferyl glucuronide, isopimpinellin, 4-Methylumbelliferone sulfate, and Daphnoretin are concentrated in the ovaries of female flowers, potentially due to their antioxidant protection mechanisms during initial seed development (Baby, 2024). Studies have shown that coumarin derivatives, such as umbelliferones, bind covalently in the spore–pollen matrix on the outer wall of pollen, potentially participating in UV protection or attracting pollinating insects. Coumarins are linearly distributed in the sieve tubes of the phloem in the filaments of male flowers, and their concentration gradient is positively correlated with JA signal intensity, suggesting their involvement in hormone regulation (Wang et al., 2024; Yang et al., 2024).

Cassia bark acids are important intermediate products in the phenylpropanoid metabolic pathway that are involved in lignin synthesis and defense responses. Their distribution differences may be related to structural reinforcement and stress resistance in *A. catechu* flowers. Mass spectrometry imaging images (**Figure 3c**) showed that 2-O-p-coumaroyltartronic acid, caftaric acid–cinnamic acid, and rosmarinic acid are enriched in the perianth wall of the ovary in female flowers. As shown in previous studies, their spatial distribution patterns highly overlap with lignin deposition areas and reactive oxygen species burst sites (Zhang et al., 2023; Zhou et al., 2023), suggesting they act synergistically through two mechanisms: by enhancing the mechanical strength of the ovary via cross-linking with cell wall polysaccharides to support embryo development (Patil et al., 2020) and by maintaining redox homeostasis (Brunetti et al., 2020; Zhao et al., 2024) via enhancing hydroxyl radical scavenging. Osmanthuside A is distributed in all four locations, especially in the ovary, achieving preferential allocation of reproductive resources through a trans-organism transport system, such as phloem loading (Sun et al., 2024).

### 4.5 Exploring the potential mechanisms of metabolic regulation in *A. catechu* flowers sex differentiation

*A. catechu* L., a plant long used in traditional Chinese medicine, possesses floral structures rich in diverse bioactive compounds with demonstrated therapeutic potential. It is noteworthy that the metabolism and accumulation of these components—such as polyphenols, alkaloids, and flavonoids—may be closely associated with the development and differentiation of floral organs (Hu et al., 2023).

The metabolic regulation of sexual differentiation in areca inflorescences is intimately linked to the function and spatial distribution of particular metabolites. Jasmonic acid (JA), a key regulator of female flower development, is enriched tenfold in female flowers and distributed in a basal-high/apical-low gradient within the inflorescence. It functions by downregulating B-class floral organ identity genes, including *AGL16* and *AP3*. Furthermore, female flowers exhibit significantly elevated expression and chromatin accessibility of JA biosynthesis (e.g., *LCAT3*) and signaling genes (e.g., *JAR1*, *JAZ*) (Zhou et al., 2022). In the present study, alkaloid metabolites were found to be enriched in female flowers, potentially serving to protect ovule and seed development. Their biosynthesis may form a coordinated “gene–metabolite” regulatory module with the sex-determination region on chromosome 15, which contains genes such as *CYP703* and *AMS* (Ding et al., 2023). Additionally, elevated levels of coumarins and cassia bark acids in female flowers likely contribute to pistil protection through antioxidant activity, suggesting that these metabolites may interact with the JA pathway to constitute a metabolic network regulating sex differentiation.

## 5 Conclusion

This study utilized spatial metabolomics technology to systematically analyze the distribution characteristics of metabolites in different parts of *A*. *catechu* flowers (female flowers, male flowers, female peduncles, and inflorescences). Compared to previous metabolomic research based on HPLC/MS techniques (Song et al., 2022; Shen et al., 2017), these studies may provide precise, *in situ* information within the inflorescence of areca trees. The results indicate that key metabolites such as alkaloids, flavonoids, coumarins, and cinnamic acids exhibit significant spatial heterogeneity. Arecoline localizes specifically to the ovule and seed, suggesting a protective role in reproductive development. In contrast, flavonoids accumulate predominantly within the vascular bundles of the ovary wall, while coumarins and cassia bark acids are more abundant in female flowers, potentially contributing to antioxidant defense mechanisms during pistil development. However, the results of this study diverge from some reported alkaloid distribution patterns, which may be attributed to differences in sample sources or analytical methods. Limitations of this study include its small sample size and the resolution of spatial metabolomics, which may not suffice to unveil the cellular-level distribution of metabolites. Future research should incorporate single-cell transcriptomics techniques. When combined with flux balance analysis, this could further clarify metabolic regulatory networks. In addition, increasing the sample size and replicating the experiment will aid in verifying the findings of this study.

## Data Availability

The original contributions presented in the study are included in the article/**Supplementary Material**. Further inquiries can be directed to the corresponding author/s.
